# Locomotive Syndrome: Definition and Management

**DOI:** 10.1007/s12018-016-9208-2

**Published:** 2016-05-25

**Authors:** Kozo Nakamura, Toru Ogata

**Affiliations:** National Rehabilitation Center for Persons with Disabilities, 4-1, Namiki, Tokorozawa, Saitama Japan

**Keywords:** Locomotive syndrome, Elderly, Exercise intervention, Gait, Balance

## Abstract

Locomotive syndrome is a condition of reduced mobility due to impairment of locomotive organs. Since upright bipedal walking involves minutely controlled movement patterns, impairment of any aspect of the locomotive organs has the potential to adversely affect it. In addition to trauma, chronic diseases of the locomotive organs, which progress with repeated bouts of acute exacerbations, are common causes of the locomotive syndrome. In Japan’s super-aging society, many people are likely to experience locomotive syndrome in the later part of their lives. Exercise intervention is effective in improving motor function, but because the subjects are elderly people with significant degenerative diseases of the locomotor organs, caution should be taken in choosing the type and intensity of exercise. The present review discusses the definition, current burden, diagnosis and interventions pertaining to the locomotive syndrome. The concept and measures are spreading throughout Japan as one of the national health policy targets.

## Introduction

The average Japanese life expectancy in the year 2014 was 80.5 years for men and 86.8 years for women, higher than in the previous year. The number of Japanese people of age 65 or more in 2014 was 33 million (26.7 % of the entire population), which is the highest ever reported anywhere in the world. It is estimated that this number will reach 36.57 million (30.3 %) in 2025 [1].

This prolonged life expectancy has affected many aspects of activities of daily living among the elderly, one among which is the difficulty in locomotion. This is illustrated by a study in Kagoshima that demonstrated that issues including fear of falling (81.7 %), not being able to stand without arm support (81.1 %), not being able to ascend stairs without using rail or wall for support (81.3 %), slow gait speed (71.7 %) and refraining from going out (50 %) were common among people aged 70–74 years [2].

The locomotive system is directly responsible for mobility. The clinical practice pertaining to the locomotive systems has changed over the last 40 years owing to the higher prevalence of chronic diseases of the locomotive organs among middle-aged to elderly people [3] and markedly increased requirement for surgery for chronic diseases, in individuals over 50 years [4].

There are four key issues in clinical practice for locomotive organs, common to the geriatric population. First, acute exacerbation of diseases of the locomotive organs is often accompanied by pain, with pain in the lower extremities and back being major causes of mobility disturbance [5–9]. Second, in the presence of severe osteoporosis, procedures utilizing metal screws may not provide adequate stability and may result in specific complications [10, 11]. Third, treatment outcomes for the locomotive organ diseases in this group of patients are significantly influenced by the status of their preoperative mobility. For example, postoperative mobility following surgical operation for proximal femoral fracture is largely influenced by the patient’s preoperative mobility [12, 13], and the results of total knee arthroplasty for osteoarthritis of the knee depend on the preoperative strength of quadriceps [14, 15]. Fourth, there is an increase in the number of people whose return to their homes is delayed following orthopedic operations. This is mainly because elderly patients need a longer period of postoperative physical training to restore their mobility. Furthermore, patients who require preoperative bed rest have dramatically reduced mobility [16–18]. Difficulty in independent mobility is a risk factor for delay in discharge from the hospital [19], and motor impairments contribute to 35.1 % of cases where discharge planning is complicated. This number is much larger compared to malignant disease (16.2 %), which is the second most common cause for complicated hospital discharge [20]. These issues were not common 40 years ago.

As a part of the evolutionary process, the adaptation of vertebrates to their environment involved a change in their skeletal structure. Bipedal locomotion is a feature unique to humans [21]. Human locomotive organs have a lifespan of about 50 years, suggesting a need for additional efforts to sustain their function when used for a longer term of 80–90 years. There is evidence supporting the view that age-related movement deficits as in sit-to-stand and gait can be improved by appropriate intervention [22–31].

The Japanese Orthopaedic Association (JOA) proposed the term locomotive syndrome (locomo) in 2007, mainly to increase awareness in the society regarding this condition and its management strategies [32]. It is important that the means and purpose of management of locomotive syndrome are understood and accepted by the general population [33].

## Definition and Concept of Locomotive Syndrome

**Fig. 1:**
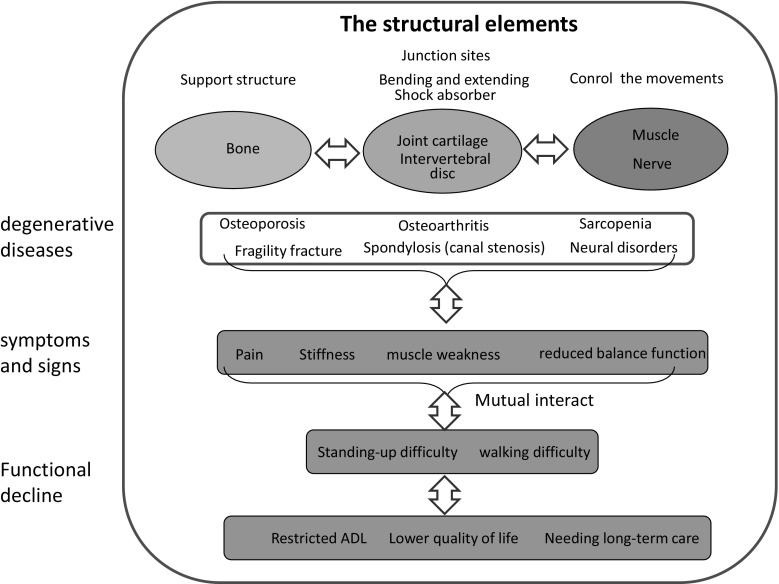
The conceptual structure of locomotive syndrome

Locomotive syndrome (locomo) is a condition wherein mobility functions such as sit-to-stand or gait are declined due to locomotive organ impairment [34]. Progression of this syndrome results in limiting independence in carrying out activities of daily living (ADL) [35]. In super-aged societies, most people experience the locomotive syndrome toward the end of their lives. Therefore, intervention is required to limit this syndrome and sustain locomotive organ function. The three main components comprising the locomotive system are bones (support), joints and intervertebral disks (mobility, impact absorption) and the muscular and nervous system (drive, control) [36, 37]. Any impairment in these organs results in pain, limited range of motion at joints or at the spine, muscle weakness and balance deficits. All these impairments are inter-related and serve as multiple risk factors for disability. Progression of these impairments eventually result in limitations in ADL, reduction of quality of life (QOL) and necessity of care support [38, 39] (see conceptual scheme in Fig. 1).

## Common Locomotive Organ Diseases

A cross-sectional study was conducted by the JOA; among new outpatients (84,544 cases) in an orthopedic clinic [40], 59.8 % had non-traumatic etiology. Among them, chronic diseases, disk degeneration (lumbar spondylosis, 11.4 %; cervical spondylosis, 4.7 %; lumbar disk hernia, 3.8 %; cervical disk hernia, 1.0 %) and lower extremity cartilage degeneration [knee osteoarthritis (OA), 6.9 %; hip OA, 1.5 %] were the most common. Among the traumatic causes, fractures of the proximal femur, which too were related to osteoporosis, were the most common (1.1 %).

## The Features of Locomotive Organ Diseases

### High Prevalence Rate

Most of the conditions contributing to the locomotive syndrome have high prevalence rates. The prevalence rates of the different conditions are as follows: Lumbar spondylosis (Kellgren–Lawrence ≥ 2) in patients above 40 years was 81.5 (males) and 65.5 % (females); knee osteoarthritis (Kellgren–Lawrence ≥ 2), 42.6 and 62.4 %; and osteoporosis [defined as femoral neck bone mineral density below the 70th percentile of young adults on dual-energy X-ray absorptiometry scan (DXA)], 12.4 and 26.5 %, in males and females, respectively [3]. The prevalence of sarcopenia was also high with rates of 13.8 % in males and 12.4 % in females [41].

### Symptoms Manifest in Subjects Over the Age of 50 Years

**Fig. 2:**
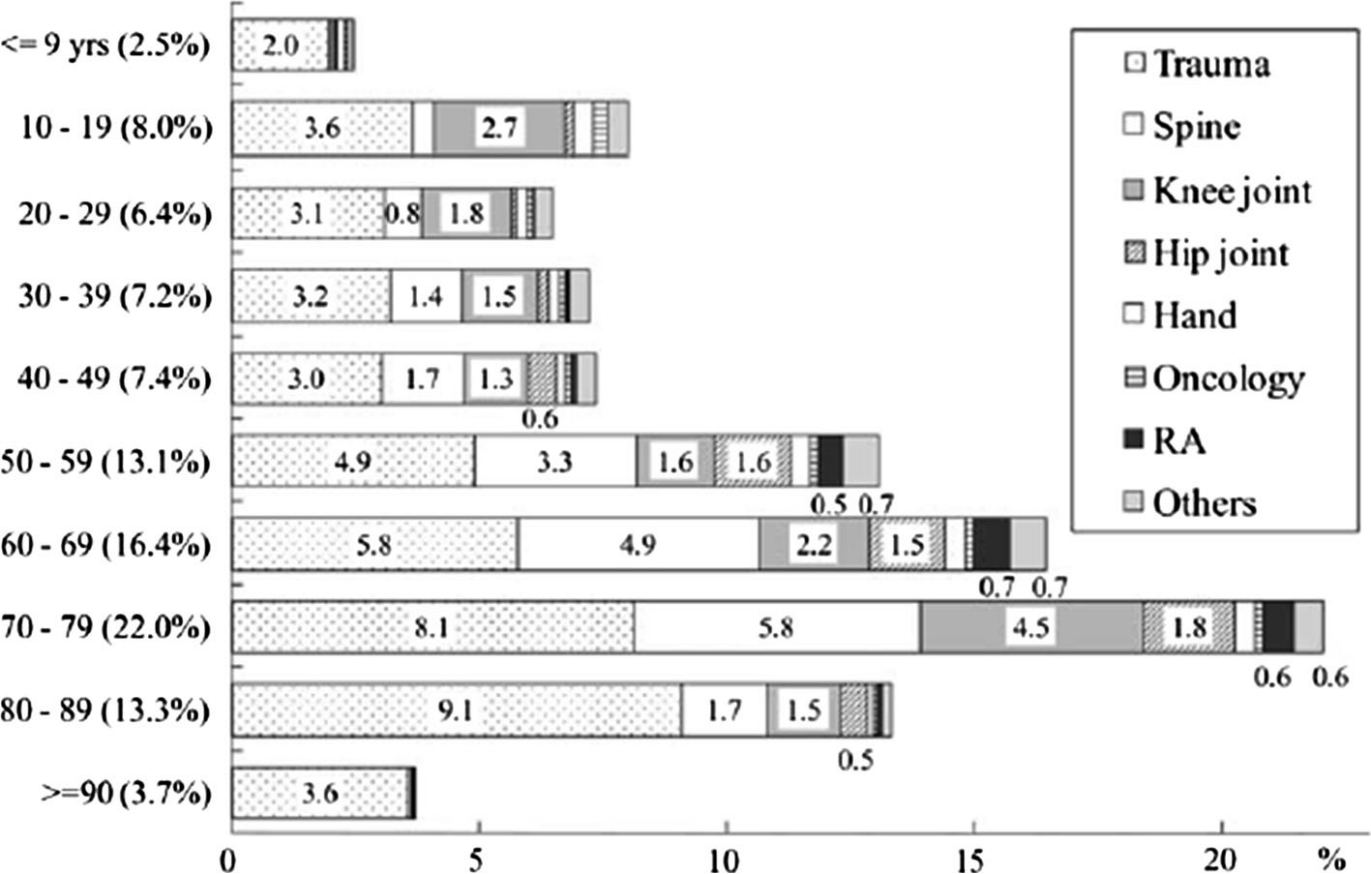
Age distribution of orthopedic surgeries [4]

In general, although locomotive degenerative diseases present with acute exacerbations, its progression in the initial stages is largely asymptomatic. The symptoms become apparent once pathological changes of degeneration become advanced, and further interventions are necessitated. The number of orthopedic surgical treatments requiring hospitalization dramatically increases after the age of 50 years (Fig. 2). The most frequent reasons for operative interventions in chronic diseases (49.7 %) were degenerated intervertebral disk (16.6 %; spondylosis, spinal canal stenosis, disk hernia), knee OA (7.1 %) and hip OA (5.4 %). Trauma accounted for the remaining 46.3 % of all cases requiring operations, most commonly for hip fractures (18.4 %) [4].

### Risk of Impairments is Different for Bone/Muscle and Joint/Intervertebral Disk

The risk of impairment varies between the different tissues that are affected. Insufficient loads and extreme thinness are the risk factors for osteoporosis [42–44] and sarcopenia [45–47] affecting bones and muscles, respectively. On the other hand, excessive loading and obesity are the risk factors for deformation and impairment of joints and intervertebral disks [36, 48–50]. The load on joints tends to be concentrated on the articular cartilage and intervertebral disks since these are mobile structures that are designed to absorb impacts. Moreover, these tissues lack direct blood supply and, thus, have minimal potential to regenerate [51–53]. Therefore, joints and intervertebral disks commonly wear out over time with aging and become painful by the middle or elderly years, when they require exercise interventions [3].

## Assessments

Degenerative changes in the locomotive components (bone, joint, muscle and nerve) result in decline in mobility. Although many tools have been developed to assess mobility, each method of assessment is designed for specific purposes. This variation in the purpose of the assessment makes it difficult to select an optimal tool [54]. Therefore, adequate care should be employed in the choice of an appropriate assessment tool with reference to why, where and how it is to be used [55, 56].

Early detection of symptoms and examination findings are important for early intervention and prevention of progression of the chronic diseases. Disability is defined as experienced difficulty in performing activities [57], and therefore, activities of daily living (ADL) and instrumental activities of daily living (IADL) are often used as assessment tools [23, 58].

Tobimatsu [59] used the 25-question Geriatric Locomotive Function Scale (GLFS-25) [60] as an assessment scale for difficulty and disability in daily activities related to locomotive organs and investigated the order of questionnaire items. This was done by stratifying the frequency of the people who had difficulties in accomplishing the task in each item. The results of this study suggested that people developed difficulties in IADL items earlier than in ADL items. Moreover, mild difficulties in going up- and downstairs, walking briskly and long-distance walking (more than 2–3 km), along with body pain (upper/lower extremities, back or neck), were experienced before the deficits in IADL or social functions were noted. In addition, most subjects expressed anxiety about being unable to walk in the future. These results are consistent with other previous studies that reported earlier onset of deficits with IADL items than with ADL items [61, 62]. This data highlight the importance of detecting minor changes in difficulty for IADL items [63, 64]. It is also important to recognize that restrictions and decline in life-space mobility may be early signs of increasing vulnerability to disability [65–69].

In Japan, the long-term care insurance system was started in 2000 to provide daily supports for elderly people. The reduction in the number of people requiring this service is one of the targets of the national health policy. Physical dysfunction in daily living (WOMAC function score, men ≥ 5, women ≥ 4), an ADL-related factor, was identified as a risk factor for certified need of care within a 4-year interval in community residents aged over 65 years [70]. Grip strength, knee extension torque, usual gait speed, chair stand time and muscle dysfunction (defined by the European Working Group on Sarcopenia in Older People algorithm for screening sarcopenia) were identified as factors determining physical function [71]. The results of these studies indicate the importance of sit-to-stand and gait function assessment.

To enable widespread acceptance among all subjects at risk, which comprises a large number, the assessment methods should be accessible to the population [33], feasible as self-tests [72, 73] and subject to easy and unambiguous interpretation, in addition to guiding disease management. Therefore, the JOA introduced a battery of short tests for recognizing patients with locomotive syndrome. These include “stand-up test,” “two-step test” and “25-question Geriatric Locomotive Function Scale (25-question GLFS)” [74].

### Short Test Battery for Locomotive Syndrome [74]

#### Stand-Up Test (Fig. 3)

The knee extensor strength of the quadriceps femoris muscle is widely used as an assessment of lower extremity muscle strength. Weight-bearing index (WBI), as an indicator of lower extremities strength, is calculated by normalizing the knee extensor strength by the body weight [75, 76]. WBI of ≥0.4 is required for normal gait, and ≥0.6 is required for independent ADL and for performing exercises such as jogging. Muranaga [77] demonstrated that the ability to stand up from a 40-cm-high stool with single-leg stance and a 20-cm-high stool with a double-leg stance could be used as screening methods to confirm WBI of ≥0.6 and ≥0.4, respectively.

**Fig. 3:**
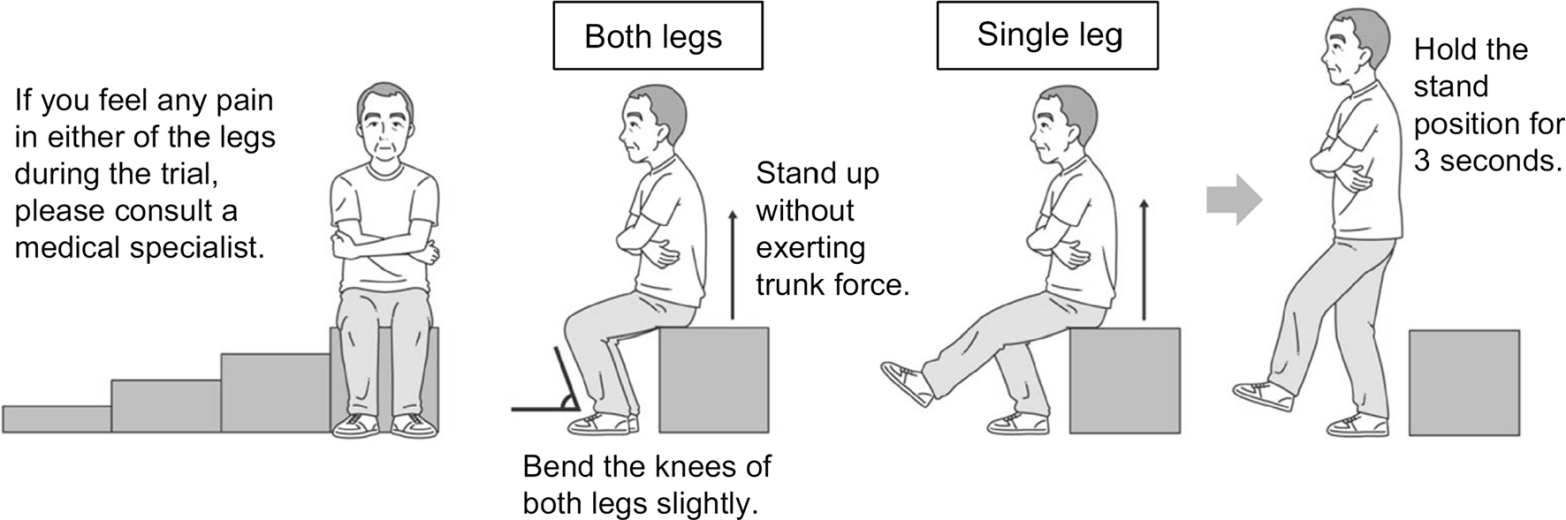
Stand-up test [74]

In the screening test, the ability to stand with a single- or double-leg stance from stools of heights, 40, 30, 20 and 10 cm, is evaluated. The grading of difficulty, from easy to difficulty, is in the order of double-leg stance with 40, 30, 20 and 10 cm stools, followed by single-leg stance with 40, 30, 20 and 10 cm. The test result is expressed as the minimum height of the stool that the subject was able to stand up from. The stand-up movement requires adequate range of motion at the joint, flexibility and balance, in addition to lower extremity muscle strength.

#### Two-Step Test (Fig. 4)

For assessment of gait-related parameters, gait speed [78–80] and maximal step length (MSL, the ability to maximally step out and return to the initial position [81]) are used. MSL is recognized as a useful tool for evaluation of balance and can be performed within a small space [73, 82, 83]. The two-step test score is calculated by normalizing the maximal length of two steps taken by the subject, by the subject’s height. This test was developed by Muranaga for assessment of gait function [84]. This test has the ability to detect bilateral impairment, and the movement pattern assessed is similar to the actual gait of the subject [72]. The test results are easy to interpret and positively correlate with maximal gait speed [85].

**Fig. 4:**
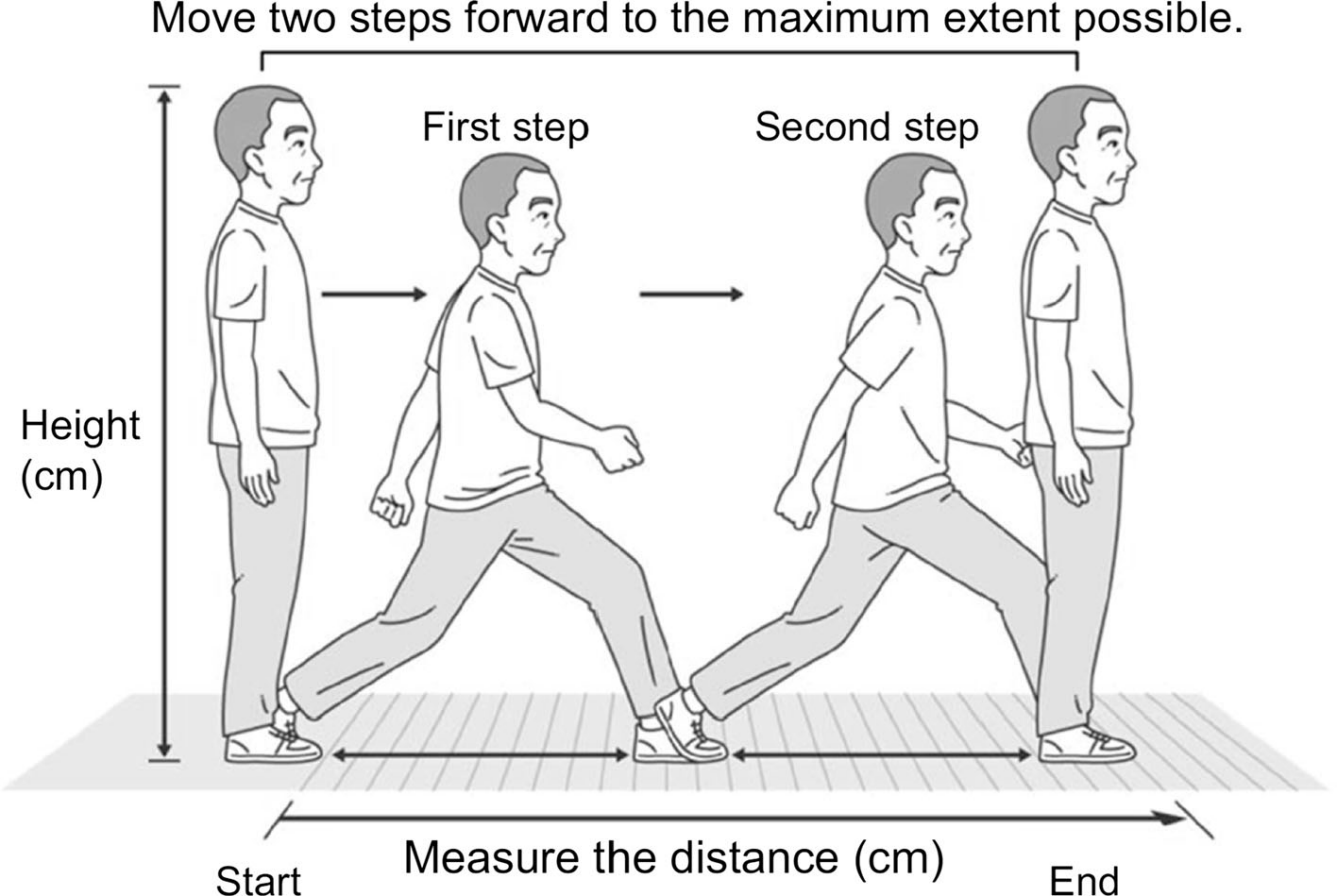
Two-step test [74]

#### 25-Question GLFS

The importance of self-rated evaluation for physical function and health status is well known [26, 57, 86]. Seichi et al. [60] developed the 25-question GLFS as an assessment tool for early detection of locomotive syndrome. The scale is a self-reported comprehensive measure, consisting of 25 questions referring to the preceding month. The scale includes four questions regarding pain, 16 questions regarding activities of daily living, three questions regarding social functions, and two questions regarding mental health status. Each item is graded on a five-point scale, from no impairment (0) to severe impairment (4 points), and the total score is derived by the sum of all scores (minimum = 0, maximum = 100). The total score is assumed to represent a quantitative evaluation of the difficulties and disabilities in daily life activity related to locomotive organs. The age-specific mean values are 5.8 in the 40s, 6.0 in the 50s, 5.9 in the 60s and 8.8 in the 70s [87]. People with a score ≥16 are expected to have limitations in walking and going out [60].

### Clinical Decision Limits for Assessing the Risk of Locomotive Syndrome [34, 88]

The JOA proposed clinical decision limits of these three tests as a guide to assessing the risk of locomotive syndrome. In their proposal, clinical decision limits were established in two stages.

#### Stage 1

The following criteria indicate a beginning of the decline of mobility function, and the subject is categorized as Stage 1 if any of the three conditions are met.

Stand-up test, difficulty in one-leg standing from a 40-cm-high seat (either leg).

- Two-step test, <1.3.
- 25-question GLFS score, ≥7.

Subjects categorized in Stage 1 are recommended to perform exercise training (locomotion training) (vide infra).

#### Stage 2

The following criteria indicate a progression of the decline of mobility function, and the subject is categorized as Stage 2 if any of the three conditions are met.

Stand-up test, difficulty in standing from a 20-cm-high seat using both legs.

- Two-step test, <1.1.
- 25-question GLFS score, ≥16.

Subjects categorized in Stage 2 need to perform exercise training. In the presence of pain, medical consultation is recommended since it may be an indicator of underlying pathological changes in locomotive organs.

### Relationship Between Clinical Decision Limits and Mobility Function

Yoshimura et al. [88] evaluated the feasibility of the clinical decision limit values by analyzing their relationship with decline in mobility functions (gait speed <0.8 m/s [78–80], five times sit-to-stand test time >12 s [89]) in community residents. They demonstrated that in both Stages 1 and 2, the clinical decision limit values based on the three tests correlated with the decline in mobility functions. In addition, the odds of decline in mobility functions exponentially increased with the increase in the number of criteria fulfilled.

### The Number of People with Locomotive Syndrome

The current estimated number of people above 40 years categorized as Stage 1 is 45.9 million (males, 20.2 million; females, 25.7 million) and as Stage 2 is 13.8 million (males, 4.6 million; females, 9.2 million) (unpublished data).

## Locomotion Training

### Physical Interventions for Mobility Function

Many studies have reported the effectiveness of physical intervention in limiting the disability and functional decline of mobility, strength, balance and gait in geriatric population [22–28, 31, 90]. In general, while physical interventions are effective in people with mild to moderate disability [23, 58], their utility is limited in people with severe disability [25], emphasizing the importance of early detection of the locomotive syndrome and early intervention. In addition, which physical interventions are the most effective remains unclear [90, 91].

Physical interventions are based on the principles of exercise [92]. First, it is known that the particular body components or skills, which are involved in a given exercise, will demonstrate improvement (principle of specificity). Second, a high load is required for any functional improvement (principle of overload). Third, it is important to gradually increase the exercise load (principle of progression) with consideration for safety since the majority of the middle- to old-aged population have chronic degeneration of intervertebral disks or lower limb cartilages such as in the knee joint [3].

Given these conditions, locomotion training, called locotra, aims to improve and sustain standing and gait functions in middle- and old-aged subjects, by recommending squatting and single-leg standing with eyes open [34, 93]. These exercises are recommended as they are directly related to standing, and gait functions [91] are safe and are feasible at home for self-management [33].

### Locomotion Training (Locotra) [93]

#### Single-Leg Standing with Eyes Open (Fig. 5)

This balance exercise, single-leg standing with eyes open, can be done alone [94] or combined with other muscle power training (like chair-rising training) [95]. This test has been demonstrated to be effective in preventing falls.

**Fig. 5:**
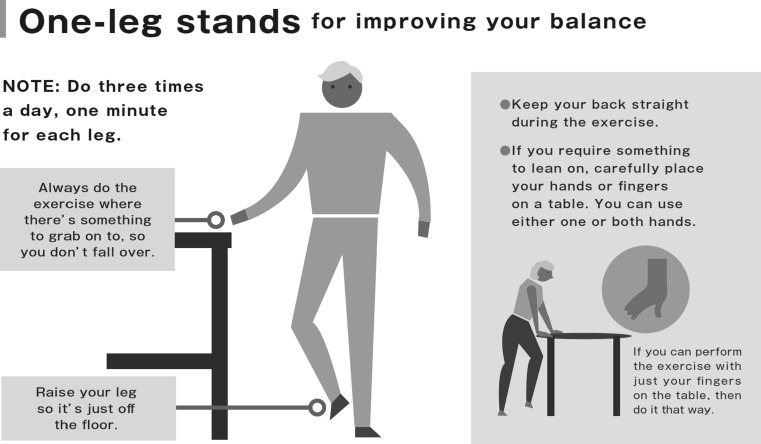
Single-leg standing with eyes open. Locomotive syndrome pamphlet. https://locomo-joa.jp/en/index.pdf

The training involves subjects standing on one leg with their eyes open for 1 min. Subjects are instructed to perform this by standing adjacent to a stable chair or desk for arm support, to prevent from falling. The exercise performed for each leg at a time constitutes one set. Subjects are recommended to perform 3 sets each in the morning, noon and evening, every day.

#### Squatting (Fig. 6)

Previous studies have demonstrated the effectiveness of squatting in improving independence of ADL, in addition to strength and balance of lower limb and body [96, 97].

**Fig. 6:**
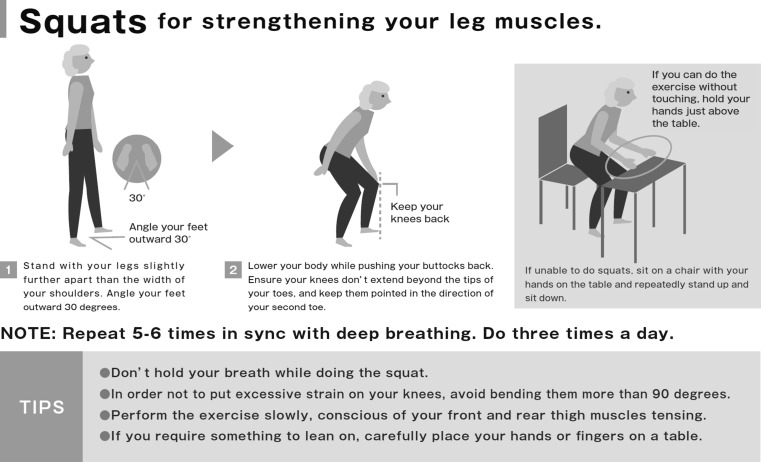
Squatting. Locomotive syndrome pamphlet. https://locomo-joa.jp/en/index.pdf

Subjects slowly move the torso down from the standing position as is done during stand–sit movement. Subjects are instructed to maintain the position of the patella (knee) over the toes in order to prevent overload on the knee. The knee flexion angle should not exceed 90°. One set comprises of 5–6 slow squats, and about three sets are to be performed each day.

#### Management in People with Mild Locomotive Syndrome

Walking is recommended [67, 98–100]. The number of repetition of the basic locotra is increased, and other exercises, such as heel raise and front lunges, are added.

### Examples of Locotra-Intervention

In Niigata, Aoki et al. [101] recruited 97 community-dwelling adults (age, 76.8 ± 5.8 years; males, 29; females, 68) who did not participate in the government-sponsored prevention programs. The prevalence of locomotor symptoms was high among the recruited subjects: low back pain in 69.1 %, knee pain in 57.7 % and osteoporosis in 35.1 % of subjects. Participants received locomotion training (one-leg standing with eyes open and squatting) instruction and performed exercises independently for 3 months as monitored by using serial telephonic calls. Among the recruited subjects, 87 (89.7 %) completed the intervention. Scores from physical function tests (single-leg standing and five times sit-to-stand tests), and seven of eight SF-8 subscales were significantly improved. Low back pain was alleviated in 12.6 % and worsened in 2.3 %, while knee pain was alleviated in 17.2 % and worsened in 1.1 % of recruited subjects, respectively.

In Saitama, Ishibashi and Fujita recruited 151 females (age, 76.6 ± 5.6 years) who participated in a health lecture meeting [102]. Several of these women had diagnostic history of locomotive diseases: knee osteoarthritis in 61.1 %, lumbar spinal stenosis in 38.7 % and osteoporosis in 46.4 %. Participants received locomotion training (one-leg standing with eyes open and squatting) instruction and performed exercises independently for 2 months. Among the recruited subjects, 97 (64.2 %) completed the intervention. Scores from physical function tests (one-leg standing on the left side, 10 m maximal gait speed, knee extension torque) improved significantly following the intervention.

In Yamagata, 60 subjects (females, 45; males, 15; mean age, 76.3 ± 5.8 years), who did not attend the on-site preventive care programs of the long-term care insurance system, participated in an intervention program. Several of the included subjects had history of locomotor symptoms: low back pain, 56.7 %; knee pain, 73.3 %; and osteoporosis, 21.7 %. Participants received locomotion training (one-leg standing with eyes open and squatting) instruction and performed exercises independently for 3 months as monitored by using serial telephonic calls. Among the recruited subjects, 55 (91.7 %) completed the intervention. Post-intervention, there was a significant improvement in one-leg standing time. Subjects who practiced squatting more often (mean, 2.82 sets/day) were more likely to be in the highly improved group (one-leg standing time ≥9.50 s) compared to those who practiced squatting lesser (*p* = 0.04) [103].

## Discussion

Impaired mobility is a major problem in Japan’s super-aged society. Physical performance is composed of multiple components including muscular strength, endurance, flexibility, balance, speed, reaction time and power. Therefore, the tools used for the assessment of mobility should be carefully selected after considering the purpose and utility of the results of assessment. In view of the magnitude of the problem, it should also be recognized that the feasibility of assessment methods is an important factor in preventive management of diseases with high prevalence rates [33, 55, 56].

In Japan’s super-aged society, it is common to encounter middle- and old-aged people in the community who walk or ascend/descend stairs with difficulty. This situation is more serious in clinical practice, with a high incidence of fractures in the elderly, caused mainly owing to unstable sit-to-stand or gait. In addition, refraining from going out due to knee pain contributes to social disability. From the clinical point of view, sit-to-stand and gait functions are fundamental for daily living. The motivating factors in proposing the locomotive syndrome were to enable early detection of people with declined sit-to-stand and gait functions, and early intervention as a means of improving these functions. For this to be achieved, it is important that the general population comprehends the purpose and means of management of locomotive syndrome.

The locomotive training method is multifaceted and incorporates exercises to improve balance and strengthen muscles. These include chair-rising, squats, Tai Chi, dance, walking and their combinations [22, 24, 27, 104]. However, as of now, it is unclear as to which method is the best [90, 91]. Highly effective training requires the performance of high-intensity exercises. On the other hand, safety considerations are important, especially in the middle- and old-aged population. In fact, a U-shaped correlation between exercise intensity and improvement of function in the geriatric population has been demonstrated [105, 106]. In addition, the results of the studies reviewed by us proved that locotra, comprising only low-intensity, short-duration exercises, was effective. Hashimoto et al. [103] reported that the effectiveness of training was directly proportional to the frequency of training, suggesting the importance of regular and consistent training.

Pain is an important factor contributing to impairment of the locomotor organs and is a major cause of movement disorders in humans [7, 8, 107, 108]. All the three studies discussed by us included participants with high prevalence of locomotor symptoms requiring intervention [16]. All the studies reported significant benefits with an exercise intervention program, and no adverse effects were reported [101].

The persistence rate of participants in the exercise intervention program tended to be higher when supported by serial telephonic communication [101, 103], compared to instances where there was no such support [102]. This suggests that serial telephonic support may be an effective means of ensuring compliance with the exercise program [109]. Studies have confirmed the importance of community support in ensuring the success of the exercise intervention program.

Studies documenting the benefits of locotra have certain limitations, namely none of the studies are randomized controlled trials, the duration of intervention is short and limited to a few months, and the follow-up is inadequate. In addition, analysis is based only on cross-sectional data. These limitations need to be addressed by future studies.

The concept of locomotive syndrome is gaining popularity in Japan [110]. The National Health Promotion program of Japan (2013–2022), titled “Health Japan 21 (second term),” which targets achieving an extension of healthy life expectancy, specifically aims to increase the recognition of locomotive syndrome from its present level of 17.3–80 % among the population above the age of 20 years [111]. As in April 2016, this figure has improved to 47.3 % [112]. In May 2015, a special issue was published titled, “All about Locomotive Syndrome” and distributed to doctors in all departments, which indicates that locomotive syndrome is now regarded as a theme of lifetime education for medical doctors [34]. Moreover, the concept of locomotive syndrome has been included as a part of community health promotion in Fukuoka [113] and Kagoshima prefectures [114], and Kyoto [115] and Yokohama cities [116].

In conclusion, the concept of locomotive syndrome is gaining traction in the community, and it is important to further promote awareness and to educate the population at risk as a means of extending the gains made so far.
